# ‘Minimal symptom expression’ in patients with acetylcholine receptor antibody-positive refractory generalized myasthenia gravis treated with eculizumab

**DOI:** 10.1007/s00415-020-09770-y

**Published:** 2020-03-18

**Authors:** John Vissing, Saiju Jacob, Kenji P. Fujita, Fanny O’Brien, James F. Howard, The REGAIN study group, Claudio Gabriel Mazia, Miguel Wilken, Fabio Barroso, Juliet Saba, Marcelo Rugiero, Mariela Bettini, Marcelo Chaves, Gonzalo Vidal, Alejandra Dalila Garcia, Jan De Bleecker, Guy Van den Abeele, Kathy de Koning, Katrien De Mey, Rudy Mercelis, Délphine Mahieu, Linda Wagemaekers, Philip Van Damme, Annelies Depreitere, Caroline Schotte, Charlotte Smetcoren, Olivier Stevens, Sien Van Daele, Nicolas Vandenbussche, Annelies Vanhee, Sarah Verjans, Jan Vynckier, Ann D’Hont, Petra Tilkin, Alzira Alves de Siqueira Carvalho, Igor Dias Brockhausen, David Feder, Daniel Ambrosio, Pamela César, Ana Paula Melo, Renata Martins Ribeiro, Rosana Rocha, Bruno Bezerra Rosa, Thabata Veiga, Luiz Augusto da Silva, Murilo Santos Engel, Jordana Gonçalves Geraldo, Maria da Penha Ananias Morita, Erica Nogueira Coelho, Gabriel Paiva, Marina Pozo, Natalia Prando, Debora Dada Martineli Torres, Cristiani Fernanda Butinhao, Gustavo Duran, Tomás Augusto Suriane Fialho, Tamires Cristina Gomes da Silva, Luiz Otavio Maia Gonçalves, Lucas Eduardo Pazetto, Luciana Renata Cubas Volpe, Luciana Souza Duca, Maurício André Gheller Friedrich, Alexandre Guerreiro, Henrique Mohr, Maurer Pereira Martins, Daiane da Cruz Pacheco, Luciana Ferreira, Ana Paula Macagnan, Graziela Pinto, Aline de Cassia Santos, Acary Souza Bulle Oliveira, Ana Carolina Amaral de Andrade, Marcelo Annes, Liene Duarte Silva, Valeria Cavalcante Lino, Wladimir Pinto, Natália Assis, Fernanda Carrara, Carolina Miranda, Iandra Souza, Patrícia Fernandes, Zaeem Siddiqi, Cecile Phan, Jeffrey Narayan, Derrick Blackmore, Ashley Mallon, Rikki Roderus, Elizabeth Watt, Stanislav Vohanka, Josef Bednarik, Magda Chmelikova, Marek Cierny, Stanislava Toncrova, Jana Junkerova Barbora Kurkova, Katarina Reguliova, Olga Zapletalova, Jiri Pitha, Iveta Novakova, Michaela Tyblova, Ivana Jurajdova, Marcela Wolfova, Henning Andersen, Thomas Harbo, Lotte Vinge, Susanne Krogh, Anita Mogensen, John Vissing, Joan Højgaard, Nanna Witting, Anne Mette Ostergaard Autzen, Jane Pedersen, Juha-Pekka Erälinna, Mikko Laaksonen, Olli Oksaranta, Tuula Harrison, Jaana Eriksson, Csilla Rozsa, Melinda Horvath, Gabor Lovas, Judit Matolcsi, Gyorgyi Szabo, Gedeonne Jakab, Brigitta Szabadosne, Laszlo Vecsei, Livia Dezsi, Edina Varga, Monika Konyane, Giovanni Antonini, Antonella Di Pasquale, Matteo Garibaldi, Stefania Morino, Fernanda Troili, Laura Fionda, Francesco Saccà, previous, Alessandro Filla, sub-investigators, Teresa Costabile, Enrico Marano, Angiola Fasanaro, Angela Marsili, Giorgia Puorro, Renato Mantegazza, Carlo Antozzi, Silvia Bonanno, Giorgia Camera, Alberta Locatelli, Lorenzo Maggi, Maria Pasanisi, Angela Campanella, Amelia Evoli, Paolo Emilio Alboini, Valentina D’Amato, Raffaele Iorio, Maurizio Inghilleri, Laura Fionda, Vittorio Frasca, Elena Giacomelli, Maria Gori, Diego Lopergolo, Emanuela Onesti, Vittorio Frasca, Maria Gabriele, Akiyuki Uzawa, Tetsuya Kanai, Naoki Kawaguchi, Masahiro Mori, Yoko Kaneko, Akiko Kanzaki, Eri Kobayashi, Hiroyuki Murai, Katsuhisa Masaki, Dai Matsuse, Takuya Matsushita, Taira Uehara, Misa Shimpo, Maki Jingu, Keiko Kikutake, Yumiko Nakamura, Yoshiko Sano, Kimiaki Utsugisawa, Yuriko Nagane, Ikuko Kamegamori, Tomoko Tsuda, Yuko Fujii, Kazumi Futono, Yukiko Ozawa, Aya Mizugami, Yuka Saito, Makoto Samukawa, Hidekazu Suzuki, Miyuki Morikawa, Sachiko Kamakura, Eriko Miyawaki, Hirokazu Shiraishi, Teiichiro Mitazaki, Masakatsu Motomura, Akihiro Mukaino, Shunsuke Yoshimura, Shizuka Asada, Seiko Yoshida, Shoko Amamoto, Tomomi Kobashikawa, Megumi Koga, Yasuko Maeda, Kazumi Takada, Mihoko Takada, Masako Tsurumaru, Yumi Yamashita, Seiko Yoshida, Yasushi Suzuki, Tetsuya Akiyama, Koichi Narikawa, Ohito Tano, Kenichi Tsukita, Rikako Kurihara, Fumie Meguro, Yusuke Fukuda, Miwako Sato, Meinoshin Okumura, Soichiro Funaka, Tomohiro Kawamura, Masayuki Makamori, Masanori Takahashi, Namie Taichi, Tomoya Hasuike, Eriko Higuchi, Hisako Kobayashi, Kaori Osakada, Tomihiro Imai, Emiko Tsuda, previous, Shun Shimohama, Takashi Hayashi, Shin Hisahara, Tomihiro Imai, Jun Kawamata, Takashi Murahara, Masaki Saitoh, Shun Shimohama, Shuichiro Suzuki, Daisuke Yamamoto, Yoko Ishiyama, Naoko Ishiyama, Mayuko Noshiro, Rumi Takeyama, Kaori Uwasa, Ikuko Yasuda, Byung-Jo Kim, Chang Nyoung Lee, Yong Seo Koo, Hung Youl Seok, Hoo Nam Kang, HyeJin Ra, Byoung Joon Kim, Eun Bin Cho, MiSong Choi, HyeLim Lee, Ju-Hong Min, Jinmyoung Seok, JiEun Lee, Da Yoon Koh, JuYoung Kwon, SangAe Park, Eun Haw Choi, Yoon-Ho Hong, So-Hyun Ahn, Dae Lim Koo, Jae-Sung Lim, Chae Won Shin, Ji Ye Hwang, Miri Kim, Seung Min Kim, Ha-Neul Jeong, JinWoo Jung, Yool-hee Kim, Hyung Seok Lee, Ha Young Shin, Eun Bi Hwang, Miju Shin, Anneke van der Kooi, Marianne de Visser, Tamar Gibson, Carlos Casasnovas, Maria Antonia Alberti Aguilo, Christian Homedes-Pedret, Natalia Julia Palacios, Laura Diez Porras, Valentina Velez Santamaria, Ana Lazaro, Exuperio Diez Tejedor, Pilar Gomez Salcedo, Mireya Fernandez-Fournier, Pedro Lopez Ruiz, Francisco Javier Rodriguez de Rivera, Mireya Fernandez-Fournier, Maria Sastre, Josep Gamez Carbonell, Pilar Sune, Maria Salvado Figueras, Gisela Gili, Gonzalo Mazuela, Isabel Illa, Elena Cortes Vicente, Jordi Diaz-Manera, Luis Antonio Querol Gutiérrez, Ricardo Rojas Garcia, Nuria Vidal, Elisabet Arribas-Ibar, Fredrik Piehl, Albert Hietala, Lena Bjarbo, Ihsan Sengun, Arzu Meherremova, Pinar Ozcelik, Bengu Balkan, Celal Tuga, Muzeyyen Ugur, Sevim Erdem-Ozdamar, Can Ebru Bekircan-Kurt, Nazire Pinar Acar, Ezgi Yilmaz, Yagmur Caliskan, Gulsah Orsel, Husnu Efendi, Seda Aydinlik, Hakan Cavus, Ayse Kutlu, Gulsah Becerikli, Cansu Semiz, Ozlem Tun, Murat Terzi, Baki Dogan, Musa Kazim Onar, Sedat Sen, Tugce Kirbas Cavdar, Adife Veske, Fiona Norwood, Aikaterini Dimitriou, Jakit Gollogly, Mohamed Mahdi-Rogers, Arshira Seddigh, Giannis Sokratous, Gal Maier, Faisal Sohail, Saiju Jacob, Girija Sadalage, Pravin Torane, Claire Brown, Amna Shah, Sivakumar Sathasivam, Heike Arndt, Debbie Davies, Dave Watling, Anthony Amato, Thomas Cochrane, Mohammed Salajegheh, Kristen Roe, Katherine Amato, Shirli Toska, Gil Wolfe, Nicholas Silvestri, Kara Patrick, Karen Zakalik, Jonathan Katz, Robert Miller, Marguerite Engel, Dallas Forshew, Elena Bravver, Benjamin Brooks, Mohammed Sanjak, Sarah Plevka, Maryanne Burdette, Scott Cunningham, Mohammad Sanjak, Megan Kramer, Joanne Nemeth, Clara Schommer, Scott Tinerney, Vern Juel, Jeffrey Guptill, Lisa Hobson-Webb, Janice Massey, Kate Beck, Donna Carnes, John Loor, Amanda Anderson, Robert Pascuzzi, Cynthia Bodkin, John Kincaid, Riley Snook, Sandra Guinrich, Angela Micheels, Vinay Chaudhry, Andrea Corse, Betsy Mosmiller, Andrea Kelley, Doreen Ho, Jayashri Srinivasan, Michael Vytopil, Jordan Jara, Nicholas Ventura, Cynthia Carter, Craig Donahue, Carol Herbert, Stephanie Scala, Elaine Weiner, Sharmeen Alam, Jonathan McKinnon, Laura Haar, Naya McKinnon, Karan Alcon, Kaitlyn McKenna, Nadia Sattar, Kevin Daniels, Dennis Jeffery, Miriam Freimer, Joseph Chad Hoyle, John Kissel, Julie Agriesti, Sharon Chelnick, Louisa Mezache, Colleen Pineda, Filiz Muharrem, Chafic Karam, Julie Khoury, previous, Tessa Marburger, Harpreet Kaur, Diana Dimitrova, James Gilchrist, Brajesh Agrawal, Mona Elsayed, Stephanie Kohlrus, Angela Andoin, Taylor Darnell, Laura Golden, Barbara Lokaitis, Jenna Seelbach, Srikanth Muppidi, Neelam Goyal, Sarada Sakamuri, Yuen T. So, Shirley Paulose, Sabrina Pol, Lesly Welsh, Ratna Bhavaraju-Sanka, Alejandro Tobon Gonzalez, Lorraine Dishman, Floyd Jones, Anna Gonzalez, Patricia Padilla, Amy Saklad, Marcela Silva, Sharon Nations, Jaya Trivedi, Steve Hopkins, Mohamed Kazamel, Mohammad Alsharabati, Liang Lu, Kenkichi Nozaki, Sandi Mumfrey-Thomas, Amy Woodall, Tahseen Mozaffar, Tiyonnoh Cash, Namita Goyal, Gulmohor Roy, Veena Mathew, Fatima Maqsood, Brian Minton, H. James Jones, Jeffrey Rosenfeld, Rebekah Garcia, Laura Echevarria, Sonia Garcia, Michael Pulley, Shachie Aranke, Alan Ross Berger, Jaimin Shah, Yasmeen Shabbir, Lisa Smith, Mary Varghese, Yasmeen Shabbir, Laurie Gutmann, Ludwig Gutmann, Nivedita Jerath, Christopher Nance, Andrea Swenson, Heena Olalde, Nicole Kressin, Jeri Sieren, Richard Barohn, Mazen Dimachkie, Melanie Glenn, April McVey, Mamatha Pasnoor, Jeffery Statland, Yunxia Wang, Tina Liu, Kelley Emmons, Nicole Jenci, Jerry Locheke, Alex Fondaw, Kathryn Johns, Gabrielle Rico, Maureen Walsh, Laura Herbelin, Charlene Hafer-Macko, Justin Kwan, Lindsay Zilliox, Karen Callison, Valerie Young, Beth DiSanzo, Kerry Naunton, Michael Benatar, Martin Bilsker, Khema Sharma, Anne Cooley, Eliana Reyes, Sara-Claude Michon, Danielle Sheldon, Julie Steele, James Howard Jr, Chafic Karam, Rebecca Traub, Manisha Chopra, Tuan Vu, Lara Katzin, Terry McClain, Brittany Harvey, Adam Hart, Kristin Huynh, Said Beydoun, Amaiak Chilingaryan, Victor Doan, Brian Droker, Hui Gong, Sanaz Karimi, Frank Lin, Terry McClain, Krishna Polaka, Akshay Shah, Anh Tran, Salma Akhter, Ali Malekniazi, Rup Tandan, Michael Hehir, Waqar Waheed, Shannon Lucy, Michael Weiss, Jane Distad, Susan Strom, Sharon Downing, Bryan Kim, Tulio Bertorini, Thomas Arnold, Kendrick Henderson, Rekha Pillai, Ye Liu, Lauren Wheeler, Jasmine Hewlett, Mollie Vanderhook, Richard Nowak, Daniel Dicapua, Benison Keung, Aditya Kumar, Huned Patwa, Kimberly Robeson, Irene Yang, Joan Nye, Hong Vu

**Affiliations:** 1 Department of Neurology, Copenhagen Neuromuscular Center, Rigshospitalet, University of Copenhagen, Blegdamsvej 9, 2100 Copenhagen, Denmark; 2 Queen Elizabeth Neuroscience Centre and Wellcome Trust Clinical Research Facility, University Hospitals Birmingham NHS Foundation Trust, Mindelsohn Way, Edgbaston, Birmingham, B15 2WB UK; 3 Alnylam Pharmaceuticals, 675 West Kendall Street, Cambridge, MA 02142 USA; 4 Formerly Alexion Pharmaceuticals, 121 Seaport Boulevard, Boston, MA 02210 USA; 5 Alexion Pharmaceuticals, 121 Seaport Boulevard, Boston, MA 02210 USA; 6 Department of Neurology, University of North Carolina, 170 Manning Drive, Chapel Hill, NC 27599-7025 USA

**Keywords:** Eculizumab, Refractory, Myasthenia gravis, Minimal symptom expression, Acetylcholine receptor

## Abstract

**Background:**

The efficacy and tolerability of eculizumab were assessed in REGAIN, a 26-week, phase 3, randomized, double-blind, placebo-controlled study in anti-acetylcholine receptor antibody-positive (AChR+) refractory generalized myasthenia gravis (gMG), and its open-label extension.

**Methods:**

Attainment of ‘minimal symptom expression’ was evaluated using patient-reported outcome measures of gMG symptoms [MG activities of daily living scale (MG-ADL), 15-item MG quality of life questionnaire (MG-QOL15)] at the completion of REGAIN and during the open-label extension. ‘Minimal symptom expression’ was defined as MG-ADL total score of 0–1 or MG-QOL15 total score of 0–3.

**Results:**

At REGAIN week 26, more eculizumab-treated patients achieved ‘minimal symptom expression’ versus placebo [MG-ADL: 21.4% vs 1.7%; difference 19.8%; 95% confidence interval (CI) 8.5, 31.0; *p* = 0.0007; MG-QOL15: 16.1% vs 1.7%; difference 14.4%; 95% CI 4.3, 24.6; *p* = 0.0069]. During the open-label extension, the proportion of patients in the placebo/eculizumab group who achieved ‘minimal symptom expression’ increased after initiating eculizumab treatment and was sustained through 130 weeks of open-label eculizumab (MG-ADL: 1.7 to 27.8%; MG-QOL15: 1.7 to 19.4%). At extension study week 130, similar proportions of patients in the eculizumab/eculizumab and placebo/eculizumab groups achieved ‘minimal symptom expression’ (MG-ADL: 22.9% and 27.8%, respectively, *p* = 0.7861; MG-QOL15: 14.3% and 19.4%, respectively, *p* = 0.7531). The long-term tolerability of eculizumab was consistent with previous reports.

**Conclusions:**

Patients with AChR+ refractory gMG who receive eculizumab can achieve sustained ‘minimal symptom expression’ based on patient-reported outcomes. ‘Minimal symptom expression’ may be a useful tool in measuring therapy effectiveness in gMG.

**Trial registration:**

ClinicalTrials.gov NCT01997229, NCT02301624.

**Electronic supplementary material:**

The online version of this article (10.1007/s00415-020-09770-y) contains supplementary material, which is available to authorized users.

## Introduction

Generalized myasthenia gravis (gMG) is an autoimmune disorder characterized by muscle weakness that worsens with muscle use [1, 2]. Symptoms associated with gMG include muscle weakness resulting in dysarthria, dysphagia, dyspnoea and fatigue in the muscles of the face, neck, arms, hands and legs [3]. Although there is no generally recognized standard definition of ‘refractory’ disease in gMG, criteria for refractory disease that have been used include failure to respond to conventional treatments such as immunosuppressive therapies (ISTs), inability to reduce IST use without clinical relapse, intolerable adverse reactions to conventional treatments, requirement for large doses of potentially harmful agents such as ISTs, presence of comorbidities that contraindicate conventional treatments, requirement for repeated short-term rescue therapy (e.g. intravenous immunoglobulin and plasma exchange) and recurrent myasthenic crises [1, 4–7]. As a consequence of their continued disease symptoms and persistent morbidities, patients with refractory gMG experience a heavy clinical burden [4], which severely impairs their quality of life (QOL) [8].

More than 70% of patients with gMG produce autoantibodies directed against acetylcholine receptor (AChR); these patients are classed as being AChR+ . The presence of these antibodies leads to reduced binding of the neurotransmitter acetylcholine to its receptor, accelerated degradation of AChRs and activation of the complement cascade [9–11]. Complement activation results in the cleavage of the terminal complement protein C5 into C5a and C5b by the C5 convertase enzyme complexes, thus activating the terminal complement cascade [12]. The combination of accelerated AChR degradation and the complement cascade results in structural damage to the neuromuscular junction, contributing to impaired neurotransmission and the muscle weakness characteristic of gMG [9].

The humanized monoclonal antibody eculizumab specifically binds to and inhibits cleavage of C5 [12]. The phase 3, randomized, placebo-controlled REGAIN study demonstrated the efficacy and tolerability of eculizumab in AChR+ refractory gMG during 6 months of therapy (NCT01997229) [13]. An interim analysis of the open-label extension of REGAIN found that eculizumab remained effective and well tolerated for up to 3 years of extended treatment (NCT02301624) [14]. During these studies, key efficacy endpoint assessments included the patient-reported MG activities of daily living scale (MG-ADL) [15] and the 15-item MG quality of life questionnaire (MG-QOL15) [16].

Current definitions of minimal symptoms in MG rely on physician evaluation. There are currently no definitions of minimal symptoms based exclusively on patients’ assessments of their symptoms and QOL; this type of measurement could potentially be more meaningful for patients than physician-based evaluations. In a validation study for the MG-QOL15, patients in remission had a mean MG-QOL15 total score of 3.3 (standard deviation, 4.4), with a range of 0–15 [17]. Remission was defined as an MG composite score of 0 and a score of 0 on either the MG-ADL or the MG manual muscle test, with the exception that an eye closure score of 1 (mild weakness) was permitted [17].

For this analysis, we adapted this previous definition of remission [17] to develop the concept of ‘minimal symptom expression’, using the patient-reported measures of MG-ADL and MG-QOL15 that were used in REGAIN and the open-label extension study. This is the first analysis of its kind to use ‘minimal symptom expression’ as an efficacy endpoint in gMG.

## Methods

### Study design and participants

The efficacy and tolerability of eculizumab were assessed in a 6-month (26-week), phase 3, randomized, placebo-controlled study of patients with AChR+ refractory gMG aged 18 years or older (REGAIN) [13]. The first patient was enrolled on 30 April 2014. Eligible patients had confirmed AChR+ gMG; had an MG-ADL total score of at least 6; and had received at least two ISTs, or at least one IST with intravenous immunoglobulin or plasma exchange treatment at least four times in 12 months without symptom control. Exclusion criteria included ocular-only MG symptoms [Myasthenia Gravis Foundation of America (MGFA) class I] or myasthenic crisis at screening (MGFA class V). Full eligibility criteria have been published previously [13]. Patients could enrol in the open-label extension study in the 2 weeks after completing REGAIN to receive open-label eculizumab for up to a maximum of 4 years. The extension study was completed in January 2019 [14].

At least 2 weeks before starting study treatment, patients were vaccinated against *Neisseria meningitidis*. Patients who were not vaccinated at the appropriate time received prophylactic antibiotics until 2 weeks after vaccination. During the open-label extension study, when appropriate according to local guidelines, patients were revaccinated against *N. meningitidis*. During REGAIN, patients who previously received ISTs were required to maintain their pre-study dose and schedule. During the open-label extension of REGAIN, modifications to IST dose and schedule were permitted at the study investigator’s discretion.

All patients provided written, informed consent. Independent ethics committees or institutional review boards provided written approval for the study protocols and all amendments. The studies are registered at www.clinicaltrials.gov.

### Study treatment dosing and scheduling

During REGAIN, patients randomized to eculizumab received an induction dose of 900 mg of eculizumab on day 1 and at weeks 1, 2 and 3, followed by a maintenance dose of 1200 mg of eculizumab at week 4 and every 2 weeks thereafter [13]. Placebo was administered using the same schedule. All patients who continued into the open-label extension study from REGAIN underwent a 4-week blinded induction phase. During this phase, patients who had received eculizumab during REGAIN received eculizumab 1200 mg on day 1 and at week 2, and placebo at weeks 1 and 3 (eculizumab/eculizumab group). Patients who had received placebo during REGAIN received eculizumab 900 mg on day 1 and at weeks 1, 2 and 3 (placebo/eculizumab group). All patients received open-label eculizumab 1200 mg at week 4 and every 2 weeks thereafter.

### Assessments

The objective of REGAIN and the open-label extension study was to assess the tolerability of eculizumab and its efficacy, as measured by change in MG-ADL total score from each study’s baseline. This sub-analysis evaluated the achievement of ‘minimal symptom expression’ in both studies, defined as achievement of an MG-ADL total score of 0–1 (range 0–24) or an MG-QOL15 total score of 0–3 (range 0–60).

The proportions of patients achieving ‘minimal symptom expression’ were calculated for the eculizumab and placebo treatment groups at week 26 of REGAIN and up to week 130 of the open-label extension (a total of 156 weeks of eculizumab treatment for the eculizumab/eculizumab group and 130 weeks of eculizumab treatment for the placebo/eculizumab group). Achievement of a clinically meaningful quantitative MG (QMG) response, defined as an improvement of at least 5 points in QMG total score, during the study was also recorded.

Adverse events were reported and coded by preferred term using the Medical Dictionary for Regulatory Activities version 20.1. MG exacerbations, rescue therapy use and discontinuations because of adverse events were also recorded.

### Statistical analysis

The significance of differences between groups was evaluated by calculating *p* values based on Fisher’s exact test for categorical variables and a two-sample *t*-test for continuous variables.

## Results

### Patient demographics and characteristics

**Fig. 1:**
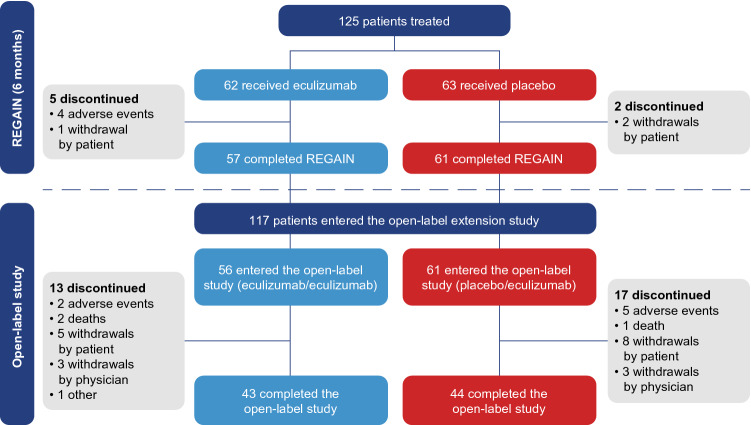
Patient disposition in REGAIN and the open-label study

Data are reported from the REGAIN study and its open-label extension for up to a maximum total of 156 weeks of eculizumab treatment. Of the 118 patients who completed REGAIN, 117 patients continued into the open-label study (eculizumab/eculizumab *n* = 56, placebo/eculizumab *n* = 61; Fig. 1) and were included in the efficacy and safety analyses. Patient demographics and characteristics were similar for the eculizumab/eculizumab and placebo/eculizumab groups, with the exception that there was a greater proportion of Asian patients in the placebo/eculizumab group (Table 1).

**Table 1:** Demographics and characteristics at REGAIN baseline of patients who continued from REGAIN into the open-label extension study

Variable	Eculizumab/eculizumab *n* = 56	Placebo/eculizumab *n* = 61	All patients *N* = 117
Age, years^a^, mean (SD)	46.8 (15.6)	47.0 (17.8)	46.9 (16.7)
Sex, *n* (%)			
Male	18 (32.1)	20 (32.8)	38 (32.5)
Female	38 (67.9)	41 (67.2)	79 (67.5)
Race, *n* (%)			
Asian	3 (5.4)	16 (26.2)	19 (16.2)
Black or African-American	0 (0.0)	2 (3.3)	2 (1.7)
White	47 (83.9)	41 (67.2)	88 (75.2)
Other/multiple/unknown	6 (10.7)	2 (3.3)	8 (6.8)
Duration of MG^b^, years, mean (SD)	10.2 (7.9)	9.2 (8.6)	9.7 (8.2)
Baseline MG-ADL total score, mean (SD)	10.3 (3.0)	9.9 (2.6)	10.1 (2.8)
Baseline MG-QOL15 total score, mean (SD)	32.5 (12.0)	30.8 (12.9)	31.6 (12.5)

*MG* myasthenia gravis, *MG-ADL* myasthenia gravis activities of daily living questionnaire, *MG-QOL15* 15-item myasthenia gravis quality of life questionnaire, *SD* standard deviation

^a^At first dose in REGAIN

^b^Time from MG diagnosis to date of first dose in REGAIN

### ‘Minimal symptom expression’ status during REGAIN

**Fig. 2:**
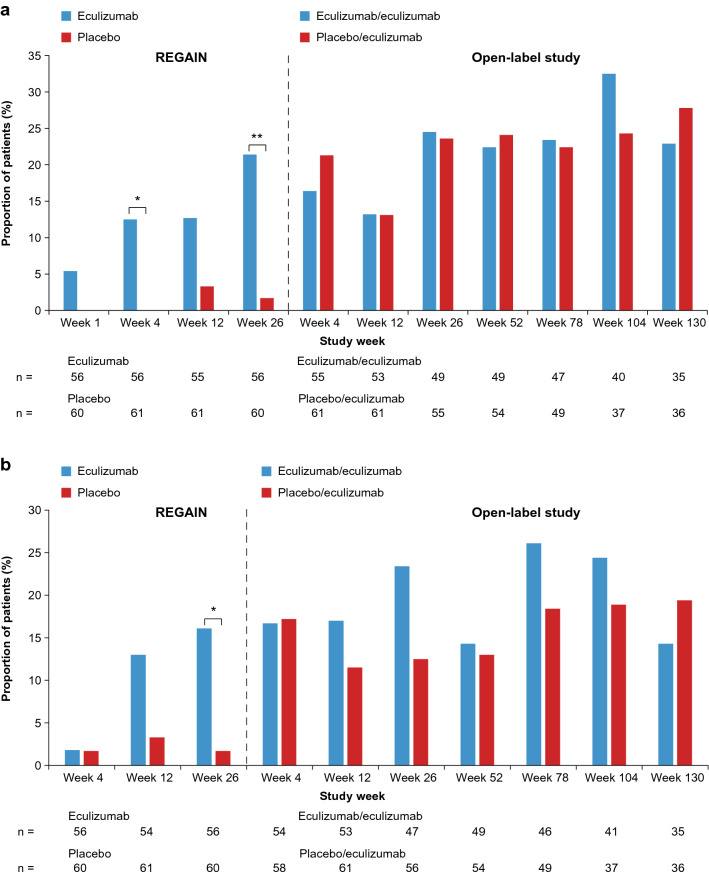
**a** Proportions of patients achieving ‘minimal symptom expression’, defined as an MG-ADL total score of 0–1. **b** Proportions of patients achieving ‘minimal symptom expression’, defined as an MG-QOL15 total score of 0–3. *p < 0.01; **p < 0.001 vs placebo. *p* values are based on Fisher’s exact test. *MG-ADL* myasthenia gravis activities of daily living questionnaire, *MG-QOL15* 15-item myasthenia gravis quality of life questionnaire

At week 26 of REGAIN, a significantly higher proportion of patients receiving eculizumab achieved ‘minimal symptom expression’ than of those receiving placebo according to MG-ADL score (21.4% and 1.7%, respectively; difference 19.8%; 95% confidence interval [CI] 8.5, 31.0; *p* = 0.0007; Fig. 2a) and MG-QOL15 score (16.1% and 1.7%, respectively; difference 14.4%; 95% CI 4.3, 24.6; *p* = 0.0069; Fig. 2b).

### ‘Minimal symptom expression’ status during the open-label study

During the open-label extension, the proportion of patients in the eculizumab/eculizumab group with ‘minimal symptom expression’ was maintained for 2.5 years, between REGAIN week 26 and open-label week 130 (MG-ADL: 21.4% and 22.9%, respectively; MG-QOL15: 16.1% and 14.3%, respectively). In the placebo/eculizumab group, the proportion of patients with ‘minimal symptom expression’ increased to levels similar to those in the eculizumab/eculizumab group in the 4 weeks after starting open-label eculizumab therapy, between REGAIN week 26 and open-label week 4 (MG-ADL: 1.7% and 21.3%, respectively; MG-QOL15: 1.7% and 17.2%, respectively). This increase was sustained to open-label week 130 (MG-ADL: 27.8%; MG-QOL15: 19.4%).

At week 130 of the open-label extension, ‘minimal symptom expression’ was achieved by similar proportions of patients in the eculizumab/eculizumab and placebo/eculizumab groups as assessed by MG-ADL score (22.9% and 27.8%, respectively; difference −4.9%; 95% CI −25.1, 15.3; *p* = 0.7861; Fig. 2a). The proportions of patients achieving ‘minimal symptom expression’ at week 130 based on MG-QOL15 score were also similar in the two groups, being 14.3% in the eculizumab/eculizumab group and 19.4% in the placebo/eculizumab group (difference −5.2%; 95% CI −22.5, 12.2; *p* = 0.7531; Fig. 2b). Overall, 25.4% of eculizumab-treated patients experienced ‘minimal symptom expression’ according to MG-ADL and 16.9% according to MG-QOL15 at this time point.

Most eculizumab-treated patients who achieved ‘minimal symptom expression’ at any time also experienced a clinically meaningful improvement in physician-reported QMG total score, defined as an improvement of at least 5 points from eculizumab start. For ‘minimal symptom expression’ according to MG-ADL total score, this proportion was 85.7% (42/49) and, for ‘minimal symptom expression’ according to MG-QOL15 total score, it was 81.1% (30/37).

There was no significant difference in mean age at first eculizumab dose between eculizumab-treated patients who achieved ‘minimal symptom expression’ according to MG-ADL at any time during REGAIN and the open-label study (up to week 130) and those who did not (47.4 vs 47.0 years; *p* = 0.8847). Mean disease duration at first eculizumab dose was shorter for patients who achieved ‘minimal symptom expression’ according to MG-ADL by open-label week 130 than for those who did not [8.27 (range 1.6–27.0) vs 11.16 (range 1.7–34.4) years; *p* = 0.0474]. For achievement of ‘minimal symptom expression’ according to MG-QOL15 up to open-label week 130, there were no significant differences in mean age (44.6 vs 48.4 years; *p* = 0.2611) or mean disease duration at first eculizumab dose [8.73 (range 1.6–24.6) vs 10.51 (range 1.7–34.4) years; *p* = 0.2091]. No significant differences were found between patients who did and did not achieve ‘minimal symptom expression’, according to either MG-ADL or MG-QOL15 scores, in other baseline characteristics, including sex, race, MGFA class, history of MG crisis and history of IST use. The only significant differences in baseline MG-ADL, MG-QOL15 and QMG total scores were for MG-ADL (*p* = 0.0380) and MG-QOL15 (*p* = 0.0487) between patients who did achieve ‘minimal symptom expression’ according to MG-QOL15 and those who did not (Table 2).

**Table 2:** Baseline demographics and characteristics of patients who did or did not achieve ‘minimal symptom expression’ at any time during REGAIN and the open-label extension study

Variable	MG-ADL total score 0–1			MG-QOL15 total score 0–3		
	Did achieve *n* = 49	Did not achieve *n* = 68	*p* value^a^	Did achieve *n* = 37	Did not achieve *n* = 80	*p* value^a^
Sex, *n* (%)						
Male	14 (28.6)	24 (35.3)	0.5491	11 (29.7)	27 (33.8)	0.8322
Female	35 (71.4)	44 (64.7)		26 (70.3)	53 (66.3)	
Race, *n* (%)						
Asian	7 (14.3)	12 (17.6)	0.5767	5 (13.5)	14 (17.5)	0.3377
Black or African American	1 (2.0)	1 (1.5)		1 (2.7)	1 (1.3)	
White	39 (79.6)	49 (72.1)		28 (75.7)	60 (75.0)	
Other/multiple/unknown	2 (4.1)	6 (8.8)		3 (8.1)	5 (6.3)	
Age at first eculizumab dose, years, mean (SD)	47.4 (18.79)	47.0 (15.25)	0.8847	44.6 (19.23)	48.4 (15.45)	0.2611
Duration of MG at first eculizumab dose^b^, years, mean (SD)	8.3 (6.57)	11.2 (9.08)	0.0474	8.7 (5.97)	10.5 (9.05)	0.2091
MG-ADL total score at REGAIN baseline, mean (SD)	9.6 (3.08)	10.4 (2.55)	0.1061	9.3 (2.79)	10.5 (2.75)	0.0380
MG-QOL15 total score at REGAIN baseline, mean (SD)	31.0 (13.23)	32.0 (12.00)	0.6709	28.2 (14.14)	33.1 (11.40)	0.0487
QMG total score at REGAIN baseline, mean (SD)	16.8 (5.51)	17.1 (5.21)	0.8247	17.1 (5.77)	16.9 (5.13)	0.9034
Patients with MGFA class at REGAIN screening, *n* (%)						
IIa	10 (20.4)	14 (20.6)	0.7087	10 (27.0)	14 (17.5)	0.7954
IIb	11 (22.4)	8 (11.8)		7 (18.9)	12 (15.0)	
IIIa	13 (26.5)	21 (30.9)		10 (27.0)	24 (30.0)	
IIIb	10 (20.4)	18 (26.5)		8 (21.6)	20 (25.0)	
IVa	2 (4.1)	4 (5.9)		1 (2.7)	5 (6.3)	
IVb	3 (6.1)	3 (4.4)		1 (2.7)	5 (6.3)	
Patients with history of MG crisis before REGAIN, *n* (%)	8 (16.3)	13 (19.1)	0.8091	6 (16.2)	15 (18.8)	0.8018
Patients using ISTs before REGAIN, *n* (%)						
1 IST	0 (0.0)	2 (2.9)	0.1818	0 (0.0)	2 (2.5)	0.0520
2 ISTs	27 (55.1)	26 (38.2)		22 (59.5)	31 (38.8)	
3 ISTs	15 (30.6)	23 (33.8)		12 (32.4)	26 (32.5)	
≥ 4 ISTs	7 (14.3)	17 (25.0)		3 (8.1)	21 (26.3)	

*IST* immunosuppressive therapy,* MG* myasthenia gravis, *MG-ADL* myasthenia gravis activities of daily living scale, *MGFA* Myasthenia Gravis Foundation of America, *MG-QOL15* 15-item myasthenia gravis quality of life questionnaire, *QMG* quantitative myasthenia gravis scale, *SD* standard deviation

^a^The significance of differences between groups was evaluated by calculating *p* values based on Fisher’s exact test for categorical variables and a two-sample *t*-test for continuous variables

^b^Time from MG diagnosis to date of first eculizumab dose

The mean MG-ADL total score for the open-label study population decreased from 10.1 [standard deviation (SD) 2.80; *n* = 117] at REGAIN baseline to 3.9 (SD 3.08; *n* = 71) at open-label week 130. The mean MG-QOL15 total score also reduced between these time points, from 31.6 (SD 12.48) to 15.3 (SD 12.15).

### Safety

Safety data have previously been published for REGAIN and an interim analysis of the open-label extension study [13, 14]. During these two studies, headache and nasopharyngitis were the most common adverse events among patients receiving eculizumab (experienced by 44.4% and 38.5%, respectively, from REGAIN baseline to week 130 of the open-label extension). MG worsening was experienced by 15.4% of eculizumab-treated patients, MG crisis by 3.4% and MG exacerbations by 29.1%. A total of 11 patients discontinued eculizumab therapy owing to adverse events during the two studies. One patient contracted a meningococcal infection, which was resolved with antibiotic treatment [13]. Three deaths were reported in patients with important comorbidities that were likely to have contributed to the clinical outcome [13].

## Discussion

This analysis found that, at the end of REGAIN, a significantly greater proportion of patients with AChR+ refractory gMG treated with eculizumab experienced ‘minimal symptom expression’ than of those receiving placebo according to an MG-ADL total score of 0–1 or an MG-QOL15 total score of 0–3. The proportions of patients experiencing ‘minimal symptom expression’ were maintained through 2.5 years of open-label eculizumab therapy in the extension study.

The only significant difference in baseline characteristics between patients who did and did not achieve ‘minimal symptom expression’ according to MG-ADL was in disease duration, and the only significant differences in the achievement of ‘minimal symptom expression’ according to MG-QOL15 were in MG-ADL and MG-QOL15 total scores at REGAIN baseline. The difference in baseline MG-ADL total score between these groups was small (1.2) and not clinically relevant. The baseline MG-QOL15 score was 4.9 points lower in patients who did achieve ‘minimal symptom expression’ according to MG-QOL15 than in those who did not, which may simply reflect that less improvement was required for patients with a lower baseline MG-QOL15 score to achieve a score of 3 or less. Overall, patients who did achieve ‘minimal symptom expression’ did not have less severe disease before eculizumab treatment than those who did not achieve it.

It is notable that, among a group of patients with refractory gMG with a mean MG-ADL total score of 10.1 at the start of REGAIN, approximately a quarter reported ‘minimal symptom expression’ defined as an MG-ADL total score of 0–1 through week 130 of the open-label study, by which time point the mean MG-ADL total score had reduced by more than half to 3.9. This reflects patient-reported improvements in disease burden in excess of the two-point reduction in MG-ADL total score that is considered to be a clinically meaningful improvement [18] to a level that has previously been described as disease remission [17]. In addition, ‘minimal symptom expression’, defined as an MG-QOL15 total score of 0–3, was achieved by one-sixth of these patients, and the mean MG-QOL15 total score halved between the start of REGAIN (31.6) and week 130 of the open-label study (15.3). The smaller proportion achieving ‘minimal symptom expression’ according to MG-QOL15 versus MG-ADL (one-sixth vs one-quarter) may be due to the conservative MG-QOL15 total score range (0–3) used in the definition of ‘minimal symptom expression’ in this analysis.

A correlation between changes in patient-reported MG-ADL scores and physician-assessed QMG scores has been described previously [19, 20]. In REGAIN and its open-label extension, patient-reported improvements were reflected in improvements in physician-reported outcomes assessed using QMG scoring. Almost half of eculizumab-treated patients achieved a clinically meaningful improvement in QMG total score (a reduction of at least 5 points) in the 26 weeks of REGAIN, and significant decreases in mean QMG total scores with eculizumab were maintained for up to 3 years during REGAIN and its open-label extension [13, 14]. In this analysis, most patients who achieved patient-reported ‘minimal symptom expression’ also achieved a clinically meaningful physician-reported QMG response.

The long-term tolerability of eculizumab was consistent with its known adverse event profile from established indications [21–25], and no new safety signals were observed since the interim analysis of the open-label extension study [14].

The main limitation of this *post hoc* analysis is the open-label design of the extension study, which could yield unconscious bias in reporting. Given that over 90% of patients who enrolled in REGAIN continued into the open-label study, selection bias in the open-label study population is unlikely. Further, the novel definition of ‘minimal symptom expression’ used in this analysis was derived from previous definitions of remission and has not yet been formally validated. In addition, further research is needed to evaluate the optimal range for this patient-reported assessment because this analysis used a conservative MG-QOL15 total score range of 0–3 to indicate ‘minimal symptom expression’.

In conclusion, the results of this analysis confirm a rapid and sustained clinical response to eculizumab in patients with refractory gMG, reflected in the higher proportion reporting ‘minimal symptom expression’ with eculizumab than with placebo. Despite having refractory MG, individuals can achieve long-term ‘minimal symptom expression’ with eculizumab therapy. The current lack of validated definitions of minimal symptoms based exclusively on patients’ assessments of their symptoms and QOL makes it difficult to comment on the generalizability of these findings. However, this type of assessment could potentially be more meaningful for patients than physician-based evaluations. ‘Minimal symptom expression’ based on quantitative, patient-reported outcomes may, therefore, be a useful tool in measuring patient progress following therapeutic intervention.

## Electronic supplementary material

Below is the link to the electronic supplementary material.

Supplementary file1 (DOCX 34 kb)

## Data Availability

Qualified academic investigators may request participant-level, de-identified clinical data and supporting documents (statistical analysis plan and protocol) pertaining to this study. Further details regarding data availability, instructions for requesting information and our data disclosure policy are available on the Alexion website (https://alexion.com/research-development).

## References

[1] Suh J, Goldstein JM, Nowak RJ (2013). Clinical characteristics of refractory myasthenia gravis patients. Yale J Biol Med.

[2] Buzzard KA, Meyer NJ, Hardy TA, Riminton DS, Reddel SW (2015). Induction intravenous cyclophosphamide followed by maintenance oral immunosuppression in refractory myasthenia gravis. Muscle Nerve.

[3] Grob D, Brunner N, Namba T, Pagala M (2008). Lifetime course of myasthenia gravis. Muscle Nerve.

[4] Engel-Nitz NM, Boscoe A, Wolbeck R, Johnson J, Silvestri NJ (2018). Burden of illness in patients with treatment refractory myasthenia gravis. Muscle Nerve.

[5] Drachman DB, Adams RN, Hu R, Jones RJ, Brodsky RA (2008). Rebooting the immune system with high-dose cyclophosphamide for treatment of refractory myasthenia gravis. Ann N Y Acad Sci.

[6] Silvestri NJ, Wolfe GI (2014). Treatment-refractory myasthenia gravis. J Clin Neuromuscul Dis.

[7] Nowak RJ, Dicapua DB, Zebardast N, Goldstein JM (2011). Response of patients with refractory myasthenia gravis to rituximab: a retrospective study. Ther Adv Neurol Disord.

[8] Boscoe AN, Xin H, L'Italien GJ, Harris LA, Cutter GR (2019). Impact of refractory myasthenia gravis on health-related quality of life. J Clin Neuromuscul Dis.

[9] Conti-Fine BM, Milani M, Kaminski HJ (2006). Myasthenia gravis: past, present, and future. J Clin Invest.

[10] Lindstrom JM, Seybold ME, Lennon VA, Whittingham S, Duane DD (1976). Antibody to acetylcholine receptor in myasthenia gravis. Prevalence, clinical correlates, and diagnostic value. Neurology.

[11] Mantegazza R, Pareyson D, Baggi F, Romagnoli P, Peluchetti D, Sghirlanzoni A (1988). Anti AChR antibody: relevance to diagnosis and clinical aspects of myasthenia gravis. Ital J Neurol Sci.

[12] Rother RP, Rollins SA, Mojcik CF, Brodsky RA, Bell L (2007). Discovery and development of the complement inhibitor eculizumab for the treatment of paroxysmal nocturnal hemoglobinuria. Nat Biotechnol.

[13] Howard JF, Utsugisawa K, Benatar M, Murai H, Barohn RJ, Illa I (2017). Safety and efficacy of eculizumab in anti-acetylcholine receptor antibody-positive refractory generalised myasthenia gravis (REGAIN): a phase 3, randomised, double-blind, placebo-controlled, multicentre study. Lancet Neurol.

[14] Muppidi S, Utsugisawa K, Benatar M, Murai H, Barohn RJ, Illa I (2019). Long-term safety and efficacy of eculizumab in generalized myasthenia gravis. Muscle Nerve.

[15] Wolfe GI, Herbelin L, Nations SP, Foster B, Bryan WW, Barohn RJ (1999). Myasthenia gravis activities of daily living profile. Neurology.

[16] Burns TM, Conaway MR, Cutter GR, Sanders DB (2008). Less is more, or almost as much: a 15-item quality-of-life instrument for myasthenia gravis. Muscle Nerve.

[17] Burns TM, Grouse CK, Conaway MR, Sanders DB (2010). Construct and concurrent validation of the MG-QOL15 in the practice setting. Muscle Nerve.

[18] Muppidi S, Wolfe GI, Conaway M, Burns TM, MG Composite and MG-QOL15 Study Group (2011). MG-ADL: still a relevant outcome measure. Muscle Nerve.

[19] Howard JF, Freimer M, O'Brien F, Wang JJ, Collins SR, Kissel JT (2017). QMG and MG-ADL correlations: study of eculizumab treatment of myasthenia gravis. Muscle Nerve.

[20] Vissing J, O'Brien F, Wang JJ, Howard JF (2018). Correlation between myasthenia gravis-activities of daily living (MG-ADL) and quantitative myasthenia gravis (QMG) assessments of anti-acetylcholine receptor antibody-positive refractory generalized myasthenia gravis in the phase 3 regain study. Muscle Nerve.

[21] Hillmen P, Young NS, Schubert J, Brodsky RA, Socie G, Muus P (2006). The complement inhibitor eculizumab in paroxysmal nocturnal hemoglobinuria. N Engl J Med.

[22] Legendre CM, Licht C, Muus P, Greenbaum LA, Babu S, Bedrosian C (2013). Terminal complement inhibitor eculizumab in atypical hemolytic-uremic syndrome. N Engl J Med.

[23] Licht C, Greenbaum LA, Muus P, Babu S, Bedrosian CL, Cohen DJ (2015). Efficacy and safety of eculizumab in atypical hemolytic uremic syndrome from 2-year extensions of phase 2 studies. Kidney Int.

[24] Socie G, Caby-Tosi MP, Marantz JL, Cole A, Bedrosian CL, Gasteyger C (2019). Eculizumab in paroxysmal nocturnal haemoglobinuria and atypical haemolytic uraemic syndrome: 10-year pharmacovigilance analysis. Br J Haematol.

[25] Zuber J, Fakhouri F, Roumenina LT, Loirat C, Fremeaux-Bacchi V, French Study Group for a HCG (2012). Use of eculizumab for atypical haemolytic uraemic syndrome and C3 glomerulopathies. Nat Rev Nephrol.

